# Supplementary material for: High KDM1A Expression Associated with Decreased CD8+T Cells Reduces the Breast Cancer Survival Rate in Patients with Breast Cancer
Source: J Clin Med. 2021 Mar 7;10(5):1112. doi: 10.3390/jcm10051112 (PMC7961911; doi:10.3390/jcm10051112)
Supplement: Supplementary file 1 [file jcm-10-01112-s001.pdf]

Supplementary Table 1. CD8+T cell immunologic gene sets within the top 10-ranked list related to KDM1A (high vs. low, TCGA).

Name	Size	ES	NES	NOM (*p*-value)	FDR (*q*-value)	FWER (*p*-value)
GSE39110 DAY3 VS DAY6 POST IMMUNIZATION CD8 TCELL DN	200	0.590	2.104	<0.001	0.001	0.033
GSE10239 NAIVE VS KLRG1HIGH EFF CD8 TCELL DN	198	0.559	2.193	<0.001	0.001	0.012
GSE39110 UNTREATED VS IL2 TREATED CD8 TCELL DAY3 POST IMMUNIZATION DN	200	0.538	2.176	<0.001	0.001	0.013
GSE10239 NAIVE VS KLRG1INT EFF CD8 TCELL DN	199	0.518	2.137	<0.001	0.001	0.024
GSE15930 NAIVE VS 24H IN VITRO STIM CD8 TCELL DN	199	0.613	2.200	<0.001	0.001	0.009
GSE15930 NAIVE VS 24H IN VITRO STIM IL12 CD8 TCELL DN	199	0.607	2.163	<0.001	0.001	0.016
GSE10239 NAIVE VS DAY4.5 EFF CD8 TCELL DN	198	0.632	2.233	<0.001	0.001	0.006
GSE19825 CD24LOW VS IL2RA HIGH DAY3 EFF CD8 TCELL DN	198	0.525	2.151	<0.001	0.001	0.018
GSE30962 ACUTE VS CHRONIC LCMV PRIMARY INF CD8 TCELL DN	200	0.566	2.159	<0.001	0.001	0.016
GSE15930 NAIVE VS 48H IN VITRO STIM IFNAB CD8 TCELL DN	198	0.599	2.122	<0.001	0.001	0.029
GSE15930 NAIVE VS 48H IN VITRO STIM IL12 CD8 TCELL DN	200	0.566	2.033	0.002	0.002	0.078
GSE15930 NAIVE VS 24H IN VITRO STIM INFAB CD8 TCELL DN	199	0.613	2.180	0.000	0.001	0.013
GSE41867 NAIVE VS EFFECTOR CD8 TCELL DN	198	0.508	2.043	0.002	0.002	0.07
GSE23568 CTRL VS ID3 TRANSDUCED CD8 TCELL DN	197	0.577	2.098	0.000	0.001	0.035
GSE15930 NAIVE VS 72H IN VITRO STIM IFNAB CD8 TCELL DN	199	0.526	2.012	0.000	0.003	0.097
GSE13493 DP VS CD4 INT CD8 POS THYMOCYTE DN	197	0.414	1.911	0.000	0.006	0.212

ES, enrichment score; NES, normalized enrichment score; FDR, false discovery rate; NOM, nominal p-value; FDR, false discovery rate; FWER, familywise error rate.

Supplementary Table 2. List of 50 breast cancer cell lines.

No.	Cell lines	KDM1A expression (z-score)	KDM1A
1	CAL-85-1	1.613	high
2	HCC1187	1.372	high
3	HCC1143	1.299	high
4	HCC2157	1.272	high
5	HDQ-P1	1.111	high
6	HCC1806	0.824	high
7	COLO-824	0.676	high
8	BT-549	0.636	high
9	BT-474	0.616	high
10	HCC1395	0.536	high
11	MDA-MB-468	0.503	high
12	MDA-MB-157	0.436	high
13	MFM-223	0.329	high
14	Hs-578-T	0.155	high
15	CAL-51	0.128	high
16	HCC1599	0.114	high
17	CAL-148	0.108	high
18	OCUB-M	0.028	high
19	HCC1937	-0.026	low
20	HCC1428	-0.18	low
21	DU-4475	-0.187	low
22	JIMT-1	-0.374	low
23	HCC70	-0.434	low
24	MDA-MB-231	-0.441	low
25	UACC-893	-0.481	low
26	EFM-19	-0.501	low
27	HCC38	-0.521	low
28	BT-483	-0.568	low
29	MDA-MB-436	-0.648	low
30	MDA-MB-361	-0.662	low
31	HCC1954	-0.682	low
32	CAMA-1	-0.715	low
33	UACC-812	-0.722	low
34	MDA-MB-330	-0.795	low
35	CAL-120	-0.963	low
36	MCF7	-0.989	low
37	HCC202	-1.016	low
38	T47D	-1.097	low
39	HCC1569	-1.137	low
40	MDA-MB-453	-1.157	low
41	BT-20	-1.277	low
42	AU565	-1.324	low
43	MRK-nu-1	-1.464	low
44	MDA-MB-415	-1.505	low
45	HCC1500	-1.585	low
46	ZR-75-30	-1.699	low
47	EFM-192A	-1.986	low
48	EVSA-T	-2.187	low
49	HCC1419	-2.675	low
50	HCC2218	-3.799	low
